# Interrogating of cancer genomes: towards more profile-based therapeutics

**DOI:** 10.1186/1753-6561-6-S6-O16

**Published:** 2012-10-01

**Authors:** John David Carpten

**Affiliations:** 1 Division of Integrated Cancer Genomics, Translational Genomics Research Institute, Phoenix, AZ, USA

## 

Although death rates due to common diseases such as heart disease, stroke and infectious diseases have declined over the last half-century, there has been only a small change in cancer mortality rates. The availability of the draft human genome sequence and a number of technological advances now provide us with the opportunity to explore the genomic landscape of cancer in an unprecedented way. Here, we will describe the application of multiple genomic technologies toward the interrogation of a number of cancer genomes, in order to discover molecular determinants of cancer that might be associated with clinical outcome and those that might be candidates for targeted therapy. It is our hope that these data would one day be translated into clinical practice to improve therapeutic decision-making for more knowledge-based clinical management.

